# Case report: Mohr-Tranebjaerg syndrome: hearing impairment as the onset of an insidious disorder with high recurrence risk

**DOI:** 10.3389/fneur.2023.1161940

**Published:** 2023-06-01

**Authors:** Eulalia Sousa, Maria Abreu, Nataliya Tkachenko, João Rocha, Cláudia Falcão Reis

**Affiliations:** ^1^Pediatrics Department, Centro Hospitalar Tâmega e Sousa, Penafiel, Portugal; ^2^Medical Genetics Unit, Centro de Genética Médica Jacinto Magalhães, Centro Hospitalar Universitário de Santo António, Porto, Portugal; ^3^Neurology Department, Centro Hospitalar Tâmega e Sousa, Penafiel, Portugal; ^4^Life and Health Sciences Research Institute (ICVS), School of Medicine, University of Minho, Braga, Portugal; ^5^ICVS/3B's-PT Government Associate Laboratory, Braga, Portugal

**Keywords:** MTS, deafness-dystonia syndrome, hearing loss, epilepsy, X-linked

## Abstract

Mohr-Tranebjaerg syndrome (MTS) is an X-linked recessive disorder caused by *TIMM8A* loss of function. It is characterized by sensorineural hearing loss in childhood, progressive optic atrophy in early adulthood, early onset dementia and psychiatric symptoms of variable expressivity. We present a family with 4 affected males, explore age-related and interfamilial variability and review the literature.

**Case report:**

A 31 years-old male developed psychiatric symptoms at age 18 and presented early onset dementia. Sensorineural hearing loss had been diagnosed in childhood. At 28yo, he developed dysarthria, dysphonia, dysmetria, limb hyperreflexia, dystonia, and spasticity following an acute encephalopathic crisis. WES revealed a hemizygous novel likely pathogenic variant in *TIMM8A*, c.45_61dup p.(His21Argfs^*^11), establishing the diagnosis of MTS. Genetic counseling of the family allowed the diagnosis of three other symptomatic relatives −3 nephews (11yo and two 6yo twins), children of a carrier sister. The oldest nephew had been followed since 4yo due to speech delay. Sensorineural hearing loss was diagnosed at 9yo, and hearing aids were prescribed. The two other nephews were monozygotic twins, and both had unilateral strabismus. One of the twins had macrocephaly and hypoplasia of the anterior temporal lobe, as disclosed by an MRI performed due to febrile seizures. Both had developmental delays, with the language being the most affected area. Their audiograms confirmed hearing loss. All three nephews were hemizygous for the familial *TIMM8A* variant.

**Discussion:**

Hearing loss, an early sign of MTS due to auditory neuropathy, can often be overlooked until more severe features of the disorder manifest. Recurrence risk is high for female carriers, and reproductive options should be offered. Early monitoring of hearing and vision loss and neurological impairment in MTS patients is mandatory since early interventions may positively impact their development. This family showcases the importance of performing a timely etiological investigation of hearing loss and its impact on genetic counseling.

## Introduction

Mohr-tranebjaerg syndrome (MTS), also known as deafness-dystonia-optic neuronopathy, is a rare X-linked recessive neurodegenerative disease characterized by pre-lingual or post-lingual sensorineural hearing impairment in early childhood, followed by slowly progressive dystonia or ataxia and optic atrophy in adolescence or adulthood (1, 2).

MTS is progressive, and other signs and symptoms may appear throughout the patient's life, such as pyramidal signs, cognitive decline, dementia, or psychiatric disorders (1–3). However, there is considerable clinical heterogeneity since both intrafamilial and interfamilial phenotypic variations have been described (4).

This syndrome is caused by hemizygous deleterious variants in *TIMM8A* or by a contiguous gene deletion at Xq22.1 encompassing *TIMM8A*. This gene encodes a small protein that localizes to the intermembrane space in mitochondria and is a component of the translational system for the import and assembly of mitochondrial inner membrane proteins (5–7). When the contiguous gene deletion includes *BTK*, located telomeric to *TIMM8A*, it additionally results in X-linked agammaglobulinemia (2, 8).

**Figure 1:**
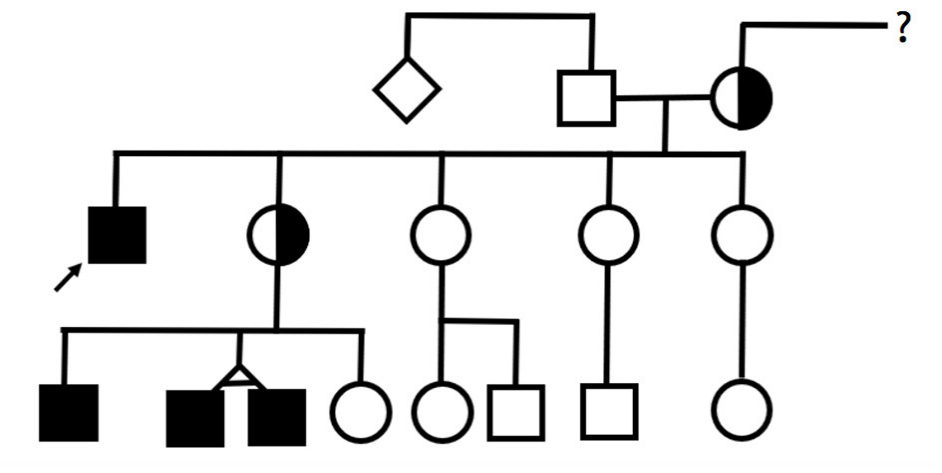
Pedigree. Filled squares represent individuals with a molecularly confirmed diagnosis of MTS.

This article presents a family with four males with MTS (Figure 1) and explores age-related and interfamilial variability. Informed consent was obtained from the mothers, the legal guardians of the patients. Institutional Review Board of Centro Hospitalar Universitário do Porto granted a waiver from review.

## Cases report

### Case 1

The index patient, a 31-year-old male, was the first child of non-consanguineous and Caucasian parents. He had four younger sisters, and there was no family history of congenital disease.

During childhood, he presented a neurodevelopmental delay, especially in language acquisition, but no motor development delay. He was diagnosed with sensorineural hearing loss from an early age and began using hearing aids. By the age of 10 years, he was diagnosed with generalized epilepsy. He maintained convulsive episodes despite being medicated in the 1st years after diagnosis, but after 16 years old had a seizure-free period of several years. He was medicated with valproic acid through adolescence and adulthood, having recently switched levetiracetam and lacosamide.

He began having severe behavioral problems in late adolescence, associated with progressive cognitive decline, and requiring several hospitalizations due to psychiatric decompensation.

At age 27, during a psychiatric hospitalization due to self and hetero-aggressiveness, he presented generalized tonic-clonic seizures and developed severe and prolonged encephalopathy with conscious level depression associated with severe nosocomial pneumonia. Non-convulsive status epilepticus and central nervous system lesions were excluded by magnetic resonance imaging (MRI). During the following weeks, with infection resolution, his conscious level improved, but his neurological state never fully recovered to the previous state. His neurological examination revealed a well-awake and aware patient with limited speech and typical dysarthria of the hearing impaired. Severe dysphagia was present during the following weeks after the encephalopathic period, and percutaneous gastrostomy was needed but removed in less than a year. He did not present any motor deficit but had a generalized increase in muscle tone, especially in the lower limbs, with moderate spasticity and hyperreflexia, with a spastic gait that was impossible without assistance. There was also slight right-hand dystonia but without functional limitation. Whenever he became severely ill by systemic disease, his neurologic deficits worsened, and there was a rapid functional decline during the following 5 years.

The following investigation was carried out: normal metabolic, endocrine, and autoimmune panels; cerebral and spinal MRI with no focal lesions or atrophy. Electromyography revealed a slight axonal type of sensory neuropathy. Mitochondrial DNA sequencing was also negative for any pathological mutation. Ophthalmologic evaluation at age 29 was normal.

As the previous etiological investigation was inconclusive, whole exome sequencing was performed and revealed a hemizygous novel likely pathogenic variant in *TIMM8A*, c.45_61dup p.(His21Argfs^*^11), establishing the diagnosis of Mohr-Tranebjaerg syndrome. This variant has not been described in the literature nor in the gnomAD and ClinVar databases.

Subsequently, the patient's family was referred for genetic counseling. The same variant was found in the mother and one of the four sisters. Genetic counseling also allowed the diagnosis of other symptomatic relatives: three nephews (cases 2–4) who were children of a carrier sister. All three nephews were hemizygous for the familial *TIMM8A* variant.

### Case 2

The oldest nephew was an 11-year-old boy, the first son of a non-consanguineous couple, born full-term after a supervised pregnancy. In the neonatal period, he performed otoacoustic emissions that were normal. At age 4, he was referred to a pediatric consultation due to a delay in developing expressive language. A global developmental delay was diagnosed, with the language area most severely affected. Afterward, behavioral problems were also reported. He started speech and occupational therapy with some progress and improvements.

At 8 years old, he underwent a cognitive assessment (Weschler Intelligence Scale for Children—WISC III) that revealed a full scale intelligence quotient (IQ) of 55, verbal IQ of 51 and performance IQ of 65.

After irregular and incomplete attendance at medical appointments and diagnostic tests, sensorineural hearing loss (70 dB bilaterally) was diagnosed at 9 years Hearing aids were prescribed, which led to a slight improvement, but without full recovery of language delay.

His neurological examination was always normal, and he had no dysmorphic features.

### Cases 3 and 4

The two other nephews were 6-year-old monozygotic twins, younger brothers of patient 2. They were born at 39 weeks, and no complications were described in the neonatal period. They performed the universal newborn hearing screening through otoacoustic emissions with a “pass” result in both ears.

Both had unilateral strabismus, one with onset in the 1st months of life and the other at around 2 years of age, with follow-up in ophthalmology.

One of the twins (case 3) was referred for a developmental consultation at 11 months due to macrocephaly (head circumference >99th percentile) and two simple febrile seizures at 7 and 10 months, without recurrence of seizures subsequently. In this context, he performed a cerebral MRI that only revealed an arachnoid cyst in the left middle fossa and associated hypoplasia of the anterior aspect of the temporal lobe. At 6 years of age, the other twin (case 4) had an episode of cervical dystonia.

Both had behavioral problems and global developmental delays. At 5 years old, they were evaluated by the Griffiths Mental Development Scale (GMDS), which revealed a general quotient of 64 and 70 in patients 3 and 4, respectively. The language subscale had the lowest developmental quotient: 38 and 52 for cases 3 and 4, respectively. The audiograms of both seem to confirm hearing loss but are non-specific due to lack of cooperation. Brainstem auditory evoked potentials while sedated have been scheduled for both.

## Discussion

MTS is characterized by a great phenotypic variability, including intrafamilial, as demonstrated by the description of these four clinical cases. Nevertheless, all these cases had in common a neurodevelopmental delay and hearing loss with onset in the first decade of life.

The hearing impairment results from auditory neuropathy. As expected, many patients with MTS have intact otoacoustic emissions, indicating normal outer hair cells, at least in the early stages of the disease, as seen in the nephews (cases 2–4) (2, 8). Hearing impairment is often the presenting manifestation, and according to the literature, it appears to be more consistent in the age of onset and progression compared to neurological, visual, and psychiatric symptoms, which vary in degree and rate of severity (2, 8). Even so, it is sometimes overlooked and underdiagnosed until more severe features of the disorder manifest. Therefore, the hearing impairment investigation is essential because an early etiological diagnosis allows for anticipating and managing other symptoms and proper family genetic counseling.

The neurological features of MTS are usually characterized by progressive movement disorder that can appear either as dystonia and ataxia, or pyramidal signs as spasticity (2, 4). The onset of dystonia and pyramidal symptoms is variable, ranging from childhood to much later (up to the sixth decade), and there is a predilection for onset in the upper limbs or craniocervical region (4, 5). The same was verified in this family with two patients who developed dystonia, one in the first decade of life and the other in adulthood but with pyramidal symptoms as the predominant picture. Some patients may also develop dysphagia, mild peripheral sensory neuropathy (2). Index patient had a slight axonal sensory neuropathy and developed periods of severe and prolonged dysphagia during a systemic infection episode.

Another characteristic of MTS is the development of neuropsychiatric symptoms. Behavioral or neuropsychiatric problems, such as mild intellectual disability, personality changes, anxiety, reduced impulse control, aggressiveness, and compromised concentration ability, may be present from childhood. Later, some patients may present paranoid psychiatric features or gradually develop dementia (2, 8). In this family, all affected males presented delayed cognitive development with developmental speech or language disorders and behavioral problems. The index case developed more severe psychiatric features and aggressive outbursts in late adolescence.

Optic neuropathy, another feature of this syndrome, may be subclinical for many years (2). Visual impairment usually manifests between the second and fourth decades of life (5). In this family, none of the affected individuals have been diagnosed with optic neuronopathy to date.

Although epilepsy has not been associated to MTS, it is a sufficient prevalent diagnosis in the general population to be a concomitant diagnosis in our index patient. Patient 3 did not develop epilepsy up to this point, having had only simple febrile seizure episodes. The authors also argue that this newly described variant could have epileptic seizures as a phenotypic manifestation. As MTS encompasses a neurodevelopment disorder, the association with epilepsy could be one to be traced in the future, as the incidence of neurodevelopmental disorders in patients with epilepsy is higher than that in the general population (9).

Genetic diagnosis is essential as it may offer opportunities for anticipatory management. The treatment of this genetic syndrome is symptomatic, consisting in managing its manifestations (8). Thus, early monitoring of hearing and vision loss and neurological impairment in MTS patients is essential since early interventions may positively impact psychomotor development and quality of life. For this purpose, regular follow-up by a multidisciplinary team is paramount.

MTS is inherited in an X-linked manner. Consequently, the recurrence risk is high for female carriers, as seen in this family. Genetic counseling should be offered to affected or at-risk individuals, including discussion of the potential risk to offspring and reproductive options. Once the pathogenic variant has been identified in the family, preimplantation genetic testing and prenatal testing are possible (2).

## Conclusion

In conclusion, Mohr-Tranebjaerg syndrome should be suspected in a family with male elements affected with hearing loss at a young age, associated with dystonia or behavioral or neuropsychiatric disorders. This family illustrates the importance of timely etiological investigation of hearing impairment and its impact on genetic counseling.

## Data availability statement

The original contributions presented in the study are included in the article/Supplementary material, further inquiries can be directed to the corresponding author.

## Ethics statement

Written informed consent was obtained from the legal guardians of the patients, for the publication of any potentially identifiable images or data included in this article.

## Author contributions

CF was the attending geneticist of the family and all MTS patients and reviewed and edited the manuscript. JR was the attending neurologist of the index patient and reviewed and edited the manuscript. NT was the initial attending geneticist of the monozygotic twin younger nephews. ES was the attending pediatrician resident. MA was the attending medical genetics resident. ES and MA drafted the manuscript. All authors revised the manuscript critically for important intellectual content and approved the final version of the manuscript.
