# Supplementary material for: Case report: Mohr-Tranebjaerg syndrome: hearing impairment as the onset of an insidious disorder with high recurrence risk
Source: Front Neurol. 2023 Jun 1;14:1161940. doi: 10.3389/fneur.2023.1161940 (PMC10267341; doi:10.3389/fneur.2023.1161940)
Supplement: Supplementary file 1 [file Data_Sheet_1.PDF]

Supplementary Material

Mohr-Tranebjaerg Syndrome: Hearing impairment as the onset of an insidious disorder with high recurrence risk

Eulalia Sousa, Maria Abreu, Nataliya Tkachenko, João Rocha, Cláudia Falcão Reis*

*** Correspondence:** Corresponding Author: claudiafalcaoreis@chporto.min-saude.pt

Supplementary Figure

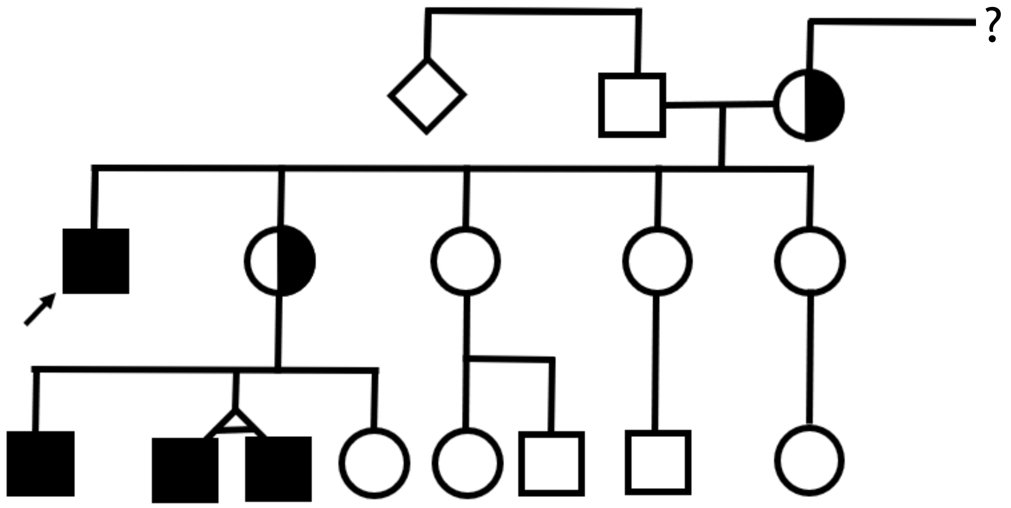

Supplementary Figure 1. Pedigree. Filled squares represent individuals with a molecularly confirmed diagnosis of MTS.
